# Circ_MUC16 attenuates the effects of Propofol to promote the aggressive behaviors of ovarian cancer by mediating the miR-1182/S100B signaling pathway

**DOI:** 10.1186/s12871-021-01517-0

**Published:** 2021-11-27

**Authors:** Hao Yang, Yunrui Guo, Yecai Zhang, Decai Wang, Guoyun Zhang, Jiali Hou, Jianming Yang

**Affiliations:** Department of Anesthesiology, The Second People’s Hospital of Kunming, Kunming College, No. 871, Longquan Road, Kunming, 650200 Yunnan China

**Keywords:** Propofol, circ_MUC16, miR-1182, S100B, Ovarian cancer

## Abstract

**Background:**

Propofol is commonly used for anesthesia during surgery and has been demonstrated to inhibit cancer development, which is shown to be associated with deregulation of non-coding RNAs (ncRNAs). The objective of this study was to explore the role of circular RNA mucin 16 (circ_MUC16) in Propofol-mediated inhibition of ovarian cancer.

**Methods:**

The expression of circ_MUC16, microRNA-1182 (miR-1182) and S100 calcium-binding protein B (S100B) mRNA was measured by quantitative real-time polymerase chain reaction (qPCR). The expression of S100B protein was checked by western blot. Cell proliferation was assessed by 3-(4, 5-di methyl thiazol-2-yl)-2, 5-di phenyl tetrazolium bromide (MTT) assay and colony formation assay. Glycolysis metabolism was assessed by glucose consumption, lactate production and ATP level. Cell migration and cell invasion were assessed by transwell assay. Cell migration was also assessed by wound healing assay. Animal study was conducted in nude mice to determine the role of circ_MUC16 in vivo. The relationship between miR-1182 and circ_MUC16 or S100B was validated by dual-luciferase reporter assay and RNA immunoprecipitation (RIP) assay.

**Results:**

Propofol inhibited ovarian cancer cell proliferation, glycolysis metabolism, migration and invasion, which were partly recovered by circ_MUC16 overexpression. Circ_MUC16 was downregulated in Propofol-treated ovarian cancer cells. Besides, circ_MUC16 knockdown enhanced the effects of Propofol to further inhibit tumor growth in vivo. MiR-1182 was a target of circ_MUC16, and circ_MUC16 knockdown-inhibited cell proliferation, glycolysis metabolism, migration and invasion were partly restored by miR-1182 inhibition. In addition, S100B was a target of miR-1182, and miR-1182-suppressed cell proliferation, glycolysis metabolism, migration and invasion were partly restored by S100B overexpression.

**Conclusion:**

Circ_MUC16 overexpression alleviated the effects of Propofol to promote the aggressive behaviors of ovarian cancer by targeting the miR-1182/S100B network.

**Supplementary Information:**

The online version contains supplementary material available at 10.1186/s12871-021-01517-0.

## Background

In 2018, there were estimated 295,414 new cases and 184,799 deaths worldwide of ovarian cancer [1]. Current statistical analysis shows that ovarian cancer is the fifth leading cause of cancer-related mortality in women worldwide, and is the most common and deadly gynecological cancer [17]. Cancer is a disease driven by multiple cellular pathways and can be affected in multiple ways [15]. According to the histological origin, ovarian cancer can be classified as epithelial, germ cell, sex cord, or interstitial tumors, and approximately 90% of primary ovarian cancers are of epithelial origin [10]. Morphological and molecular studies of ovarian cancer indicate that it should not be classified as a single disease, but should be attributed to a collection of disease subtypes with distinct origins and clinical behaviors [14, 11]. Therefore, it is necessary to explore the pathogenesis of ovarian cancer from multiple levels to improve treatment strategies.

Propofol (2, 6-diisopropylphenol) is one of the most popular intravenous anesthetics for inducing and maintaining anesthesia in modern medicine [5]. Recently, it has been reported to exhibit potential anti-cancer properties, such as inhibiting the proliferation, adhesion and metastasis of cancer cells, and inducing apoptosis [24, 18]. Further studies should be carried out to explore the role and mechanism of Propofol in cancer progression.

Previous studies disclosed that numerous non-coding RNAs (ncRNAs) were involved in Propofol-mediated cancer progression [6, 13]. Circular RNAs (circRNAs) are a kind of non-coding transcripts and have attracted much attention in recent years [4]. CircRNAs are primarily derived from precursor mRNA by back-splicing, lacking free 3′ and 5′ ends due to continuous loop structures [12]. In function, circRNAs show tremendous regulatory properties in human cancers, which provides a new perspective to understand cancer pathogenesis [26]. Circular RNA mucin 16 (circ_MUC16), also known as circ_0049116, is derived from MUC16 mRNA. Interestingly, circ_MUC16 was shown to be downregulated by Propofol in ovarian cancer cells in our analysis. We speculated that circ_MUC16 was implicated in Propofol-inhibited progression of ovarian cancer.

It is acknowledged that partial circRNAs exert their biological functions by sequestering target microRNAs (miRNAs) or RNA-binding proteins [22]. To address the potential regulatory mechanism of circ_MUC16, its targeted miRNAs were screened. MiR-1182 attracted our interest because the anti-tumor role of miR-1182 in ovarian cancer had been proposed in a previous study [9]. In this study, we explored the underlying interaction between circ_MUC16 and miR-1182. Moreover, we characterized the target genes of miR-1182 to furth er address the regulatory mechanism of circ_MUC16 because it is well known that miRNAs govern gene expression by binding to their 3′ untranslated region (3’UTR) [2]. S100 calcium binding-protein B (S100B) was a potential target of miR-1182, which was further illustrated in this study.

In the present study, we determined the effects of Propofol on ovarian cancer development and investigated the role of circ_MUC16 in Propofol-administered ovarian cancer in vitro and in vivo. Besides, we uncovered the interactions among circ_MUC16, miR-1182 and S100B, aiming to provide a potential mechanism of circ_MUC16 in Propofol-mediated ovarian cancer progression.

## Methods

### Tissues

Ovarian cancer tissues (*n* = 30) and matched normal ovarian tissues (n = 30) were collected from The Second People’s Hospital of Kunming. The use of tissues was approved by enrolled patients, and the written informed consent was obtained. Tissues were excised and frozen by liquid nitrogen, followed by storage at − 80 °C conditions. This study was approved by the Ethics Committee of The Second People’s Hospital of Kunming.

### Cells

Ovarian cancer cells (A2780 and SK-OV-3) and normal ovarian epithelial cells (IOSE-80) were purchased from Bena culture collection (Beijing, China). A2780 cells were cultured in 90% Dulbecco’s Modified Eagle Medium (DMEM) (Bena) containing 10% fetal bovine saline (FBS; Bena). SK-OV-3 cells were cultured in 90% Mc Coy’s 5a medium (Bena) containing 10% FBS. IOSE-80 cells were cultured in 90% RPMI1640 medium (Bena) containing 10% FBS. All cells were cultured in 37 °C conditions supplemented with 5% CO_2_.

### Cell treatment

Propofol was purchased from Sigma-Aldrich (St. Louis, MO, USA). Propofol was dissolved in dimethyl sulfoxide (DMSO, Sigma-Aldrich) and stored at − 20 °C. Cells were treated with different concentrations of Propofol (1, 5, 10 and 20 μg/mL) for 24 h. As for control, the cell culture medium was added the same concentration of DMSO (without propofol) that for the highest propofol concentration (20 mg/L).

### Cell transfection

Circ_MUC16 overexpression vector (circ_MUC16) and matched blank pCD-ciR vector (Vector), small interference RNA targeting circ_MUC16 (si-circ_MUC16) and matched negative control (si-NC) were all provided by Geneseed (Guangzhou, China). MiR-1182 mimic (miR-1182), miR-1182 inhibitor (anti-miR-1182) and their matched controls (miR-NC and anti-miR-NC) were bought from Ribobio (Guangzhou, China). S100B overexpression vector (S100B) and matched blank pcDNA vector (pcDNA) were constructed by Genepharma (Shanghai, China). Lipofectamine 3000 (Invitrogen, Carlsbad, CA, USA) was employed for cell transfection.

### 3-(4, 5-di methyl thiazol-2-yl)-2, 5-di phenyl tetrazolium bromide (MTT) assay

Cells were seeded into a 96-well plate (2000 cells/well) and incubated for 24 h. Then, 10 μL MTT reagent (Roche, Basel, Switzerland) was added into each well, followed by incubation for 4 h in a humidified atmosphere. Next, cells were treated with solubilization solution (Roche) to dissolve formazan. The absorbance was measured at 570 nm using a microplate reader (Thermo Fisher Scientific, Waltham, MA, USA).

### Colony formation assay

Cells were seeded into a 6-well plate (200 cells/well) and cultured in a humidified atmosphere for 12 days. The growth of colonies was observed every day. Then, colonies were fixed with paraformaldehyde (Sigma-Aldrich) and stained with crystal violet (Sigma-Aldrich). The number of colonies was counted under a light microscope (Olympus, Tokyo, Japan).

### Glycolysis detection

Glucose consumption, lactate production and ATP level were detected to assess glycolysis metabolism using matched commercial kits, including Glucose Colorimetric/Fluorometric Assay Kit (Sigma-Aldrich), Lactate Assay Kit (Sigma-Aldrich) and ATP Assay Kit (Sigma-Aldrich). All procedures were conducted according to the manufacturer’s instructions.

### Transwell assay

Transwell chambers (Corning Incorporated, Corning, NY, USA) were coated with or without Matrigel (Corning Incorporated) for invasion analysis or migration analysis, respectively. In brief, cells were resuspended in serum-free culture medium and added into the top of chambers, and culture medium containing 10% FBS was added into the bottom of chambers. After incubation for 24 h, cells failing to migrate or invade were removed using a cotton swab, and cells migrated or invade into the lower surface were fixed with paraformaldehyde (Sigma-Aldrich) and stained with crystal violet (Sigma-Aldrich). The number of cells was counted under a light microscope (100×; Olympus).

### Wound healing assay

Cells were plated into a 6-well plate until almost total confluence. Then, the monolayer was scratched using a 10 μL pipette tip and then washed with phosphate-buffered saline (PBS; Invitrogen). After incubation for 24 h, the distance of cell migration was observed under a light microscope (Olympus), and the representative images were captured at 0 h and 24 h using a light microscope (40×). Relative migration rate was investigated using Image J software (NIH, Bethesda, MA, USA).

### Quantitative real-time polymerase chain reaction (qPCR)

TRIzol reagent (Invitrogen) was utilized to isolate total RNA according to the protocol. Then, reverse transcription was performed using the PrimeScript RT reagent kit (Takara, Dalian, China) or TaqMan MicroRNA Reverse Transcription kit (Applied Biosystems, Foster City, CA, USA). Subsequently, cDNA was used for the qPCR amplification using the SYBR Green Master PCR mix (Applied Biosystems) under the ABI 7900 system (Applied Biosystems). Relative expression was normalized by β-actin or small nuclear RNA U6 and calculated using the 2^-ΔΔCt^ method. The primers used were as follows:

Circ_MUC16, F: 5′-CTCAGGCCTGTGTTCAAGAA-3′ and R: 5′-GGCCCAGCTCTTCAATGT-3′; MUC16, F: 5′-CCTCTCTGGGGACTCCATCA-3′ and R: 5′-GTCTTGGCTATGTGGGTGCT-3′; miR-1182, F: 5′-GGAGGGTCTTGGGAGGGA-3′ and R: 5′-AGTGCAGGGTCCGAGGTATT-3′; S100B, F: 5′-AGCTGGAGAAGGCCATGGTG-3′ and R: 5′-GAACTCGTGGCAGGCAGTAG-3′; β-actin, F: 5′-CTGGAACGGTGAAGGTGACA-3′ and R: 5′-CGCATCTCATATTTGGAATGACT-3′; U6, F: 5′-CTCGCTTCGGCAGCACA-3′ and R: 5′-AACGCTTCACGAATTTGCGT-3′.

### Cellular distribution analysis

Nuclear RNA and cytoplasmic RNA were extracted using the PARIS kit (Invitrogen) based on the manufacturer’s instructions. The relative expression of circ_MUC16 in several separations was detected by qPCR. β-actin and U6 were used as the internal control in cytoplasmic separation and nuclear separation, respectively.

### RNase R treatment

The isolated total RNA was treated with RNase R (2 U/μg, Epicentre, Madison, WI, USA) for 1 h at 37 °C conditions and then used for reverse transcription and qPCR.

### Animal study

Short hairpin RNA (shRNA) targeting circ_MUC16 (sh-circ_MUC16) was synthesized and packaged into a commercial lentiviral vector by Geneseed, using sh-NC as the negative control. Experimental mice (balb/c, female, *n* = 20) were purchased from Beijing Vital River Laboratory Animal Technology Co., Ltd. (Beijing, China) and divided into 4 groups (*n* = 5 per group). Mice in group 1 were subcutaneously injected with A2780 cells infected with sh-NC. Mice in group 2 were subcutaneously injected with A2780 cells infected with sh-circ_MUC16. Mice in group 3 were subcutaneously injected with A2780 cells infected with sh-NC and intraperitoneally administered with Propofol (35 mg/kg). Mice in group 4 were subcutaneously injected with A2780 cells infected with sh-circ_MUC16 and intraperitoneally administered with Propofol (35 mg/kg). During tumor growth, tumor volume (length×width^2^× 0.5) was measured every 3 days. At 22th day, tumor tissues were excised from mice for further analyses. Mice were killed by cervical dislocation after deep anesthesia with 2% isoflurane. The animal procedures were approved by the Animal Care and Use Committee of The Second People’s Hospital of Kunming.

### Dual-luciferase reporter assay

The relationship between circ_MUC16 and miR-1182 was predicted by Circular RNA Interactome (https://circinteractome.nia.nih.gov/). The relationship between miR-1182 and S100B was predicted by Targetscan (http://www.targetscan.org/vert_72/). According to the putative binding site between them, mutant sequence fragment of circ_MUC16 and S100B 3’UTR was designed. Subsequently, the wild-type and mutant-type reporter plasmids of circ_MUC16 (circ_MUC16-WT and circ_MUC16-MUT) and S100B 3’UTR (S100B 3’UTR-WT and S100B 3’UTR-MUT) were constructed by Sangon Biotech (Shanghai, China). A2780 and SK-OV-3 cells were transfected with miR-1182 or miR-NC together with circ_MUC16-WT, circ_MUC16-MUT, S100B 3’UTR-WT or S100B 3’UTR-MUT. After 48 h, luciferase activity was checked using the Dual-Luciferase Reporter Assay System (Promega, Madison, WI, USA).

### RNA immunoprecipitation (RIP) assay

Magna RNA-Binding Protein Immunoprecipitation Kit (Millipore, Billerica, MA, USA) was used to conduct RIP assay. Cell extract was incubated with RIP buffer containing magnetic beads conjugated with Ago2 antibody or negative control (IgG antibody) overnight. A protein-RNA complex was captured and dealt with proteinase K to isolate RNA fraction. Finally, RNAs eluted from beads were subjected to qPCR.

### Western blot

Total protein was obtained using Radioimmunoprecipitation assay (RIPA) buffer (Sigma-Aldrich) and quantified using a Bicinchoninic Acid (BCA) Protein Assay Kit (Millipore). Total proteins were separated using 12% sodium dodecyl sulfate-polyacrylamide gel electrophoresis (SDS-PAGE) and transferred onto polyvinylidene fluoride (PVDF) membranes (Millipore). The membranes were blocked using the Western Blocking Reagent (Roche) and then incubated with the primary antibodies, including anti-S100B (ab52642; Abcam, Cambridge, MA, USA) and anti-β-actin (ab8227; Abcam). Afterwards, the membranes were incubated with the goat-anti rabbit secondary antibody (ab205718; Abcam). The protein bands were visualized using the enhanced chemiluminescent (ECL) Detection Reagents (Beyotime, Shanghai, China).

### Statistical analysis

Data were obtained from three independent biological repeats and processed by GraphPad Prism 7 software (GraphPad Inc., La Jolla, CA, USA). The final results were shown as mean ± standard deviation (S.D.). The normally distributed data were examined by Shapiro-Wilk test, and all data satisfied the normal distribution. Difference analyses were conducted using Student’s *t*-test or analysis of variance (ANOVA) with Tukey’s test in different groups. The correlation between two sets was analyzed using Pearson correlation examination. *P* value less than 0.05 was regarded as statistical significance.

## Results

### Propofol inhibited ovarian cancer cell proliferation, glycolysis metabolism, migration and invasion

**Fig. 1:**
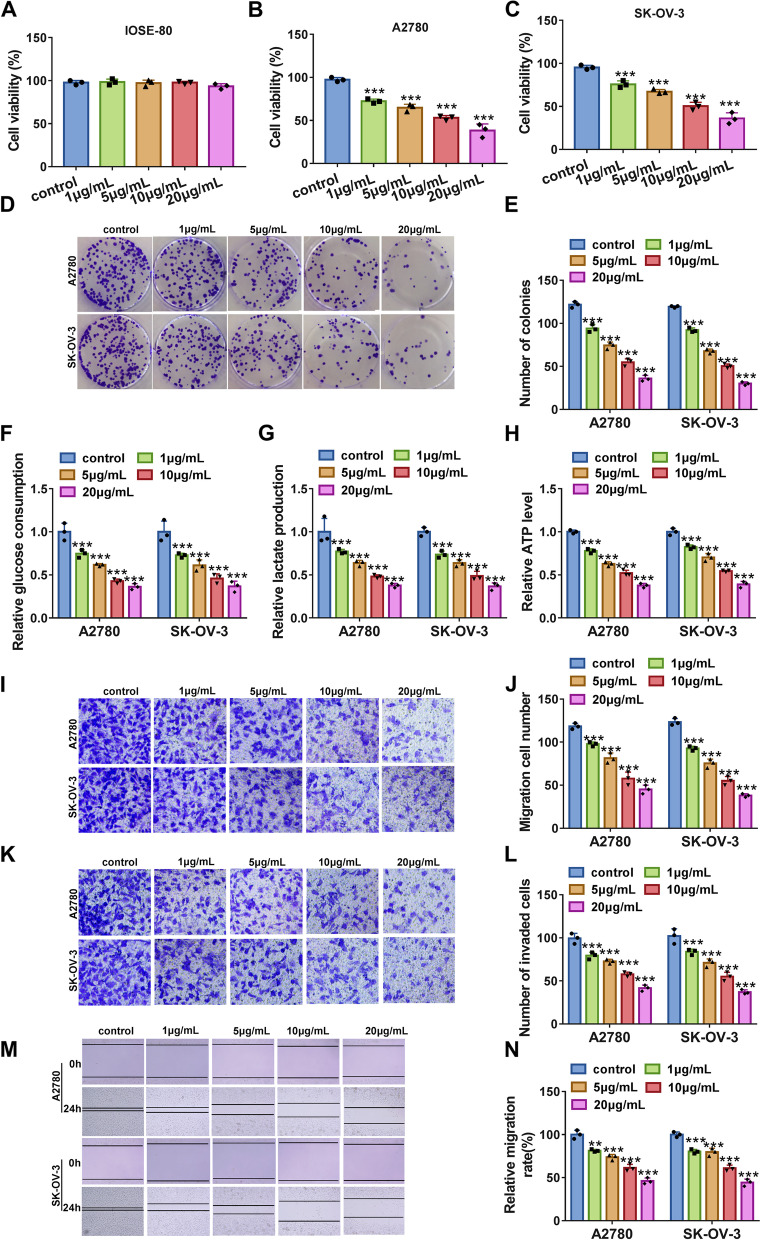
Propofol inhibited A2780 and SK-OV-3 cell proliferation, glycolysis, migration and invasion. **A**-**C** The effects of different doses of Propofol on cell viability in IOSE-80, A2780 and SK-OV-3 cells were determined by MTT assay. **D** and **E** The effects of different doses of Propofol on colony formation ability of A2780 and SK-OV-3 cells were determined by colony formation assay. **F**-**H** The effects of different doses of Propofol on glucose consumption, lactate production and ATP level in A2780 and SK-OV-3 cells were determined by matched kits. **I**-**L** The effects of different doses of Propofol on cell migration and invasion in A2780 and SK-OV-3 cells were determined by transwell assay. (M and N) The effects of Propofol on cell migration were also determined by wound healing assay. **P < 0.01 and ***P < 0.001

Non-cancer cells (IOSE-80) and ovarian cancer cells (A2780 and SK-OV-3) were treated with Propofol at different concentrations (0, 1, 5, 10 and 20 μg/mL). We found that Propofol hardly affected the viability of IOSE-80 cells (Fig. 1A), while Propofol significantly depleted the viability of A2780 and SK-OV-3 cells in a dose-dependent manner (Fig. 1B and C), suggesting that Propofol had vital effects on ovarian cancer cells. Then, we found that Propofol significantly inhibited colony formation ability of A2780 and SK-OV-3 cells in a dose-dependent manner (Fig. 1D and E). Besides, Propofol notably suppressed glucose consumption, lactate production and ATP level in A2780 and SK-OV-3 cells in a dose-dependent manner (Fig. 1F-H), suggesting that Propofol suppressed glycolysis energy metabolism in ovarian cancer cells. In addition, Propofol markedly weakened the capacities of migration and invasion in A2780 and SK-OV-3 cells by transwell assay (Fig. 1I-L), and wound healing assay also presented that the migration of A2780 and SK-OV-3 cells was blocked by Propofol (Fig. 1M and N). These data suggested that Propofol inhibited ovarian cancer progression in vitro.

### Circ_MUC16 was upregulated in tumor tissues and cell lines of ovarian cancer

**Fig. 2:**
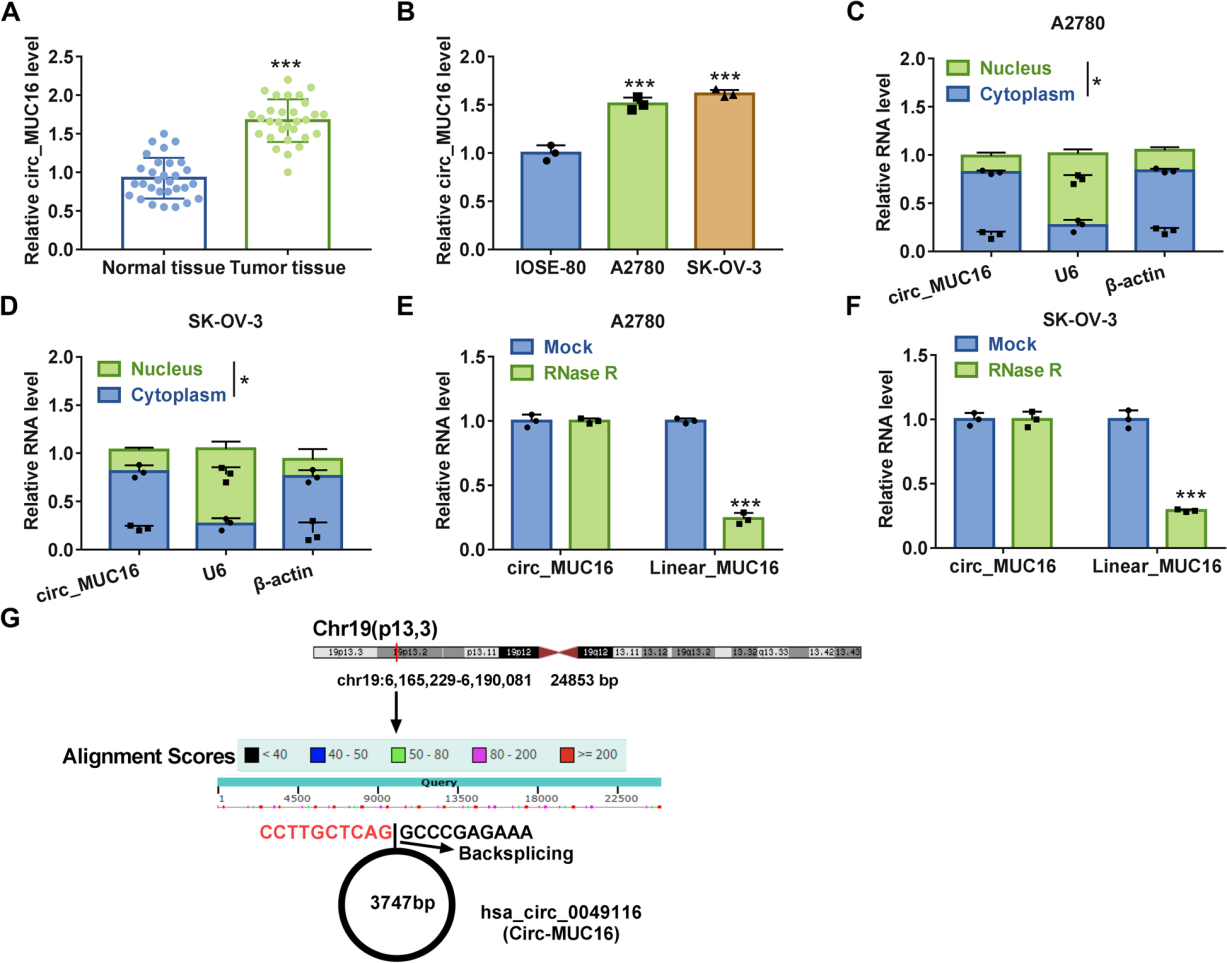
Circ_MUC16 expression was enhanced in ovarian cancer tissues and cells. **A** The expression of circ_MUC16 in tumor tissues and normal tissues was detected by qPCR. **B** The expression of circ_MUC16 in IOSE-80, A2780 and SK-OV-3 cells was detected by qPCR. **C** and **D** The location of circ_MUC16 in cytoplasm and nucleus was determined by qPCR. **E** and **F** The stability of circ_MUC16 was examined using RNase R. **G** The schematic diagram was depicted to illustrate the formation of circ_MUC16. *P < 0.05 and ***P < 0.001

Circ_MUC16 was a circRNA that was highly expressed in tumor tissues relative to matched normal tissues (Fig. 2A). Likewise, circ_MUC16 expression was obviously elevated in A2780 and SK-OV-3 cells compared with that in IOSE-80 cells (Fig. 2B). Cellular location indicated that circ_MUC16 was mainly located in the cytoplasm compared with that in the nucleus in A2780 and SK-OV-3 cells (Fig. 2C and D). RNase R treatment significantly reduced the level of linear MUC16 mRNA but hardly changed the level of circ_MUC16 in A2780 and SK-OV-3 cells (Fig. 2E and F), suggesting that circ_MUC16 was resistant to RNase R digestion. We next displayed the schematic diagram to illustrate the formation of circ_MUC16. Circ_MUC16, also known as circ_0049116, was derived from MUC16 by backsplicing (Fig. 2G). These data suggested that circ_MUC16 was dysregulated in ovarian cancer.

### Circ_MUC16 overexpression partly abolished the effects of Propofol

**Fig. 3:**
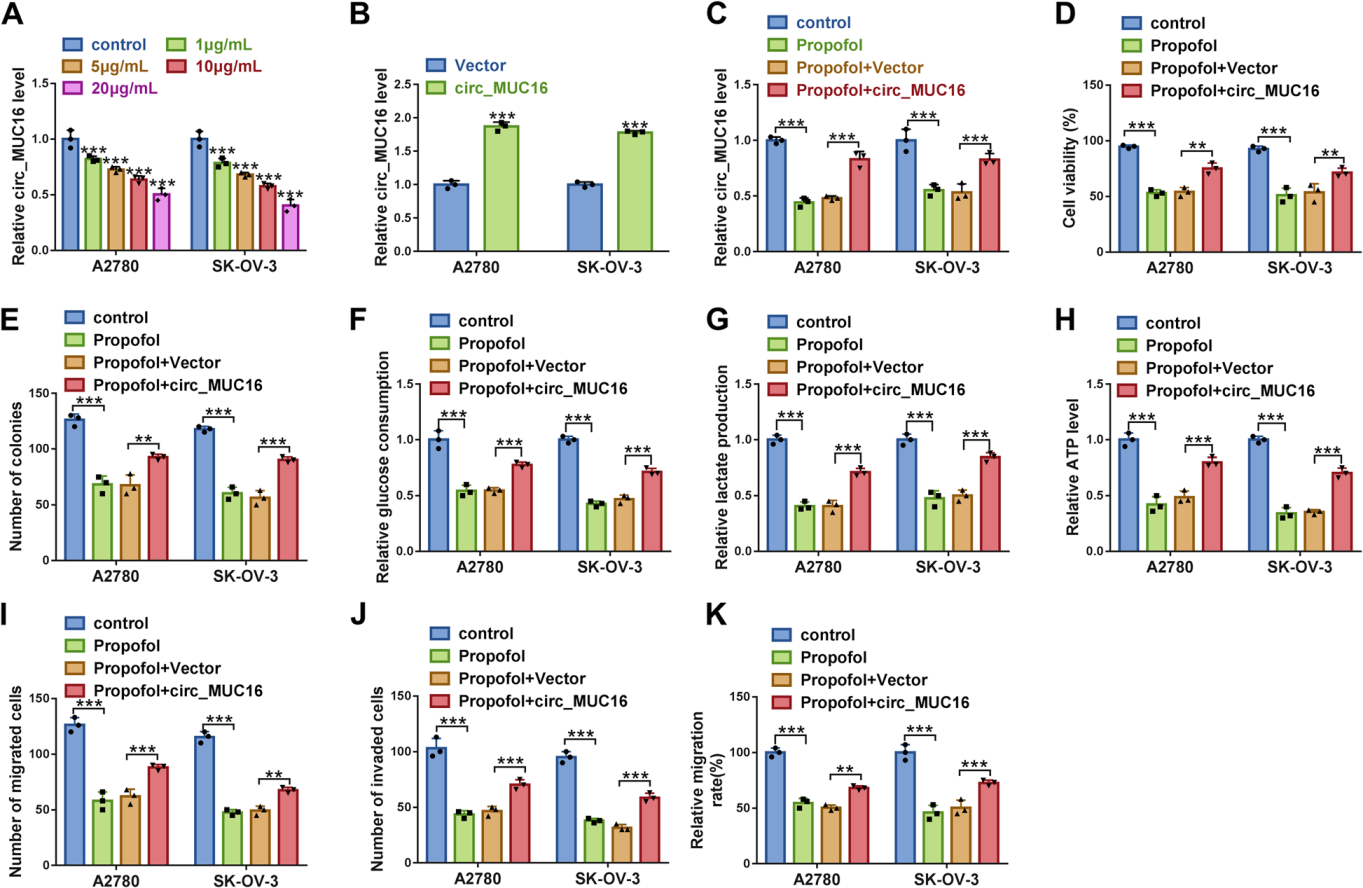
Circ_MUC16 overexpression abolished the effects of Propofol. **A** The expression of circ_MUC16 in different doses of Propofol-treated A2780 and SK-OV-3 cells was checked by qPCR. **B** The efficiency of circ_MUC16 overexpression was checked by qPCR. Then, in Propofol-treated A2780 and SK-OV-3 cells transfected with circ_MUC16 or Vector, **C** the expression of circ_MUC16 was detected by qPCR. **D** Cell viability was checked by MTT assay. **E** Colony formation ability was examined using colony formation assay. **F**-**H** Glucose consumption, lactate production and ATP level were investigated using matched kits to assess glycolysis metabolism. **I**-**J** Cell migration and cell invasion were determined by transwell assay, and **K** cell migration was also determined by wound healing assay. **P < 0.01 and ***P < 0.001

Interestingly, we found the expression of circ_MUC16 was significantly decreased in Propofol-treated A2780 and SK-OV-3 cells in a dose-dependent manner (Fig. 3A). To investigate the function of circ_MUC16, we strengthened the expression of circ_MUC16 in A2780 and SK-OV-3 cells. The expression of circ_MUC16 was significantly enhanced in A2780 and SK-OV-3 cells transfected with circ_MUC16 (Fig. 3B). Circ_MUC16 expression depleted by Propofol was largely recovered in Propofol-treated A2780 and SK-OV-3 cells transfected with circ_MUC16 (Fig. 3C). In function, Propofol-impaired cell viability of A2780 and SK-OV-3 cells was largely restored by circ_MUC16 overexpression (Fig. 3D). Propofol-inhibited colony formation ability of A2780 and SK-OV-3 cells was partly recovered by circ_MUC16 overexpression (Fig. 3E). In addition, glucose consumption, lactate production and ATP level were suppressed in Propofol-treated A2780 and SK-OV-3 cells, while glucose consumption, lactate production and ATP level were recovered in Propofol-treated A2780 and SK-OV-3 cells with circ_MUC16 overexpression (Fig. 3F-H). Moreover, the capacities of migration and invasion were inhibited by Propofol but restored by further circ_MUC16 overexpression (Fig. 3I-K). These data suggested that circ_MUC16 overexpression recovered A2780 and SK-OV-3 cell malignant behaviors that were blocked by Propofol.

### Circ_MUC16 knockdown inhibited tumor growth in vivo and strengthened the effects of Propofol

**Fig. 4:**
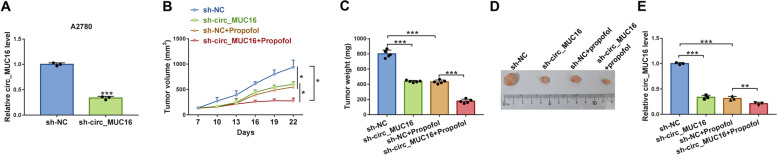
Circ_MUC16 knockdown inhibited tumor growth in vivo. **A** The efficiency of sh-circ_MUC16 was detected by qPCR. **B**-**D** Tumor volume and tumor weight were measured to assess tumor growth, and tumor size was recorded. **E** The expression of circ_MUC16 in the removed tumor tissues from different groups was detected by qPCR. **P < 0.01 and ***P < 0.001

Next, we examined the role of circ_MUC16 in vivo. A2780 cells were infected with sh-circ_MUC16 and sh-NC, and we found the expression of circ_MUC16 was notably decreased in A2780 cells infected with sh-circ_MUC16 (Fig. 4A). For tumor growth, we discovered that circ_MUC16 knockdown notably inhibited tumor volume and tumor weight, and Propofol treatment further weakened tumor volume and tumor weight in sh-circ_MUC16-infected group compared to sh-NC-infected group (Fig. 4B and C). The representative images of removed tumor nodes were shown in Fig. 4D. In the removed tumor tissues, we found that circ_MUC16 expression was strikingly declined in the sh-circ_MUC16 group and further decreased in the sh-circ_MUC16 + Propofol group (Fig. 4E). These data suggested that circ_MUC16 knockdown notably inhibited tumor growth in vivo and enhanced the effects of Propofol.

### Circ_MUC16 targeted miR-1182 and negatively regulated miR-1182 expression

**Fig. 5:**
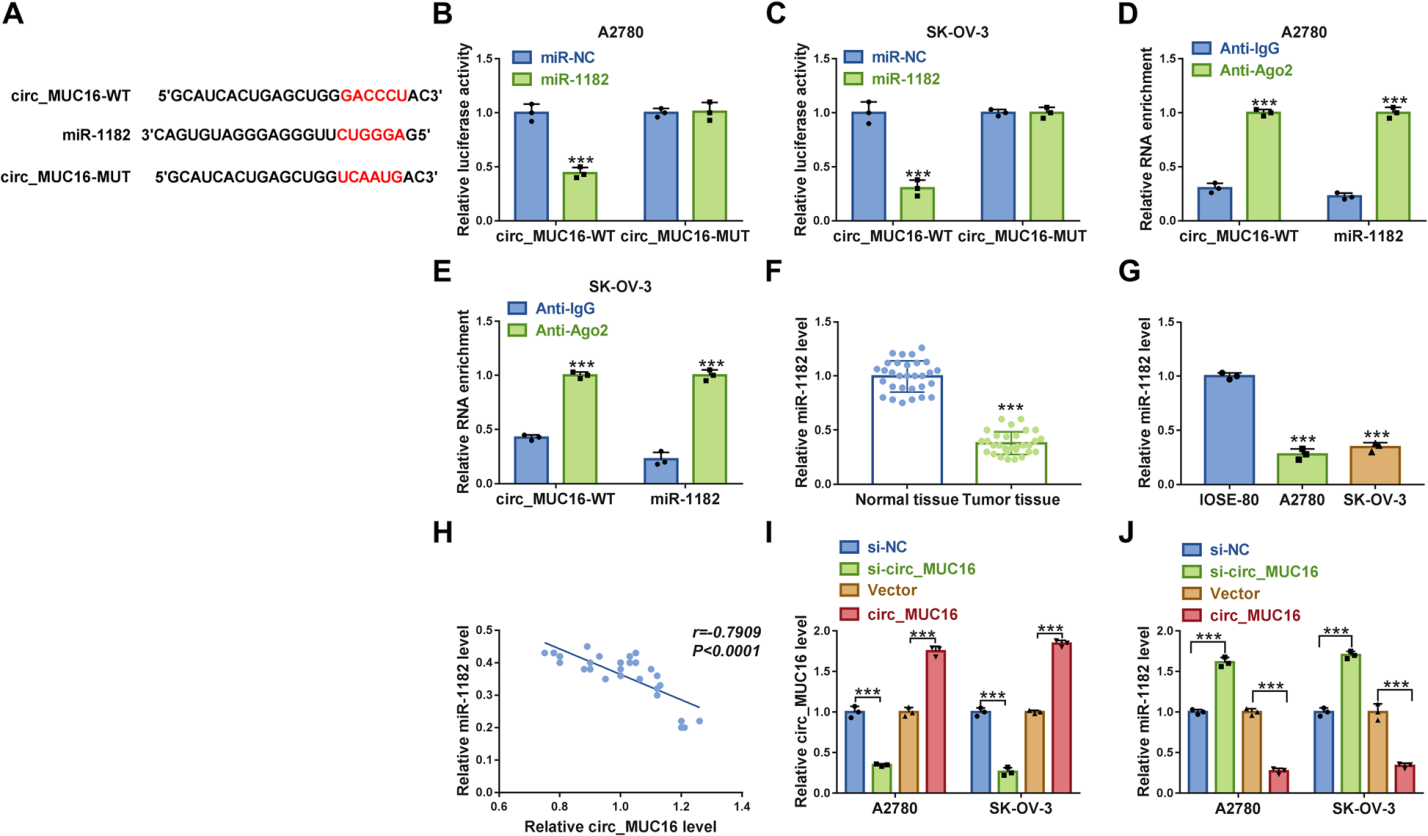
MiR-1182 was a target of circ_MUC16. **A** The relationship between circ_MUC16 and miR-1182 was predicted by Circular RNA Interactome. **B** and **C** The relationship between circ_MUC16 and miR-1182 was verified by dual-luciferase reporter assay. **D** and **E** The relationship between circ_MUC16 and miR-1182 was verified by RIP assay. **F** The expression of miR-1182 in tumor tissues and normal tissues was detected by qPCR. **G** The expression of miR-1182 in IOSE-80, A2780 and SK-OV-3 cells was detected by qPCR. **H** The correlation between miR-1182 expression and circ_MUC16 expression in tumor tissues. **I** The efficiency of circ_MUC16 knockdown or overexpression was checked by qPCR. **J** The effect of circ_MUC16 knockdown or overexpression on miR-1182 expression was checked by qPCR. ***P < 0.001

To explore the mechanism of circ_MUC16 in ovarian cancer, the potential target miRNAs of circ_MUC16 were investigated. The analysis from Circular RNA Interactome showed that miR-1182 was a target of circ_MUC16 (Fig. 5A), which was verified by dual-luciferase reporter assay and RIP assay. The data showed that the cotransfection of miR-1182 and circ_MUC16-WT significantly reduced luciferase activity in A2780 and SK-OV-3 cells (Fig. 5B and C), and both circ_MUC16 and miR-1182 could be captured by Ago2 antibody but not IgG antibody (Fig. 5D and E). Then, we observed that miR-1182 expression was declined in tumor tissues compared with that in normal tissues (Fig. 5F), and its expression was also declined in A2780 and SK-OV-3 cells compared with that in IOSE-80 cells (Fig. 5G). Besides, miR-1182 expression level was negatively correlated with circ_MUC16 expression level in tumor tissues (Fig. 5H). Moreover, after determining the efficiency of circ_MUC16 knockdown and overexpression, we found circ_MUC16 knockdown markedly increased the level of miR-1182, while circ_MUC16 overexpression markedly weakened the level of miR-1182 in A2780 and SK-OV-3 cells (Fig. 5I and J). These data suggested that circ_MUC16 acted as miR-1182 sponge to inhibit miR-1182 expression.

### MiR-1182 inhibition partly abolished the effects of Propofol

**Fig. 6:**
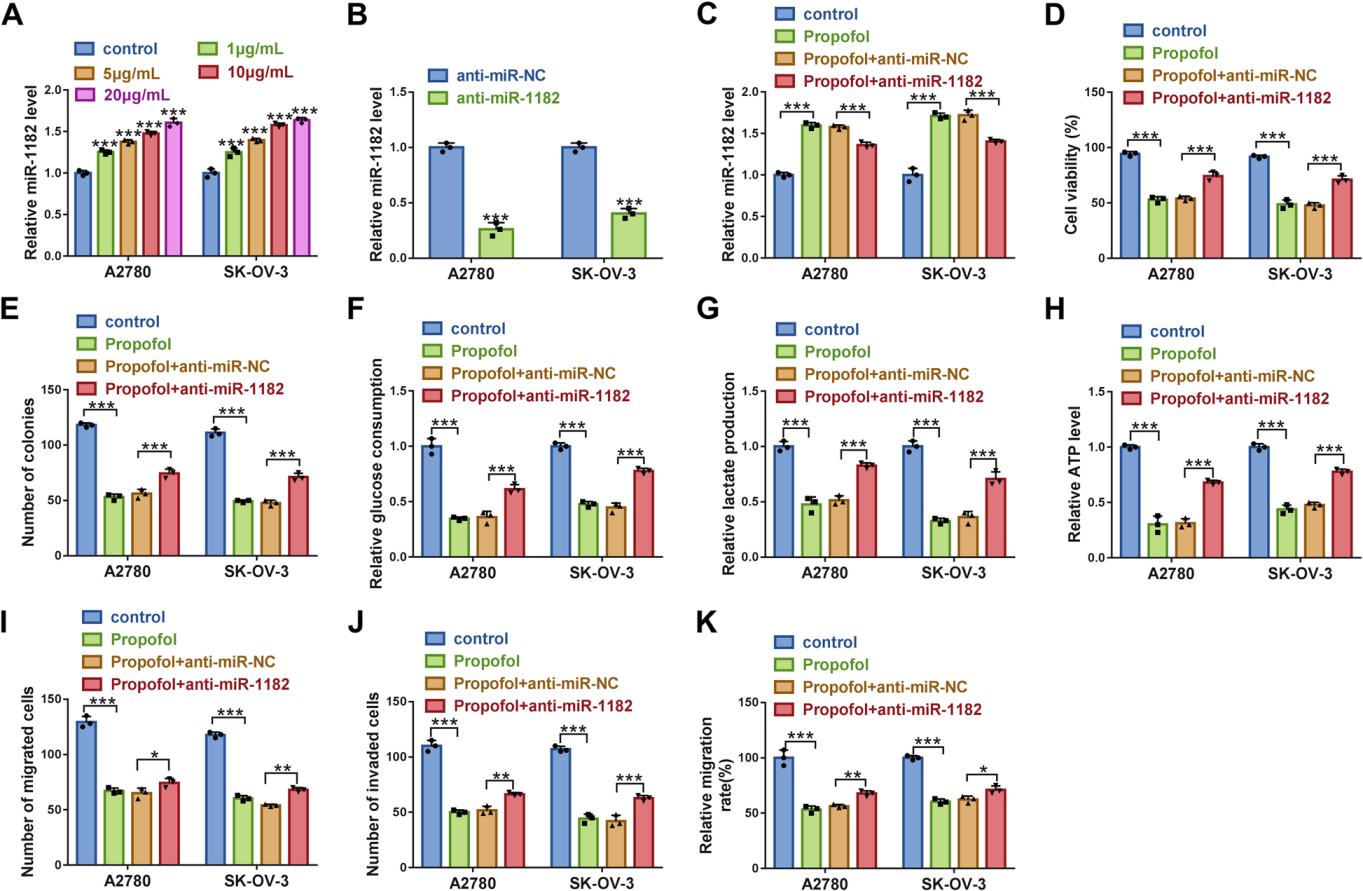
Propofol blocked A2780 and SK-OV-3 cell malignant behaviors by enriching miR-1182. **A** The expression of miR-1182 in different doses of Propofol-treated A2780 and SK-OV-3 cells was checked by qPCR. **B** The efficiency of miR-1182 inhibitor was checked by qPCR. Then, in Propofol-treated A2780 and SK-OV-3 cells transfected with anti-miR-1182 or anti-miR-NC, **C** the expression of miR-1182 was checked by qPCR. **D** Cell viability was checked by MTT assay. **E** Colony formation ability was checked by colony formation assay. **F**-**H** Glycolysis consumption, lactate production and ATP level were measured using matched kits. **I**-**K** Cell migration and invasion were assessed by transwell assay, and cell migration was also assessed by wound healing assay. *P < 0.05, **P < 0.01 and ***P < 0.001

The data from qPCR displayed that the expression of miR-1182 was significantly increased in Propofol-treated A2780 and SK-OV-3 cells in a dose-dependent manner (Fig. 6A). The efficiency of miR-1182 inhibitor was examined, and we found that miR-1182 expression was notably declined in A2780 and SK-OV-3 cells transfected with anti-miR-1182 compared to anti-miR-NC (Fig. 6B). Then, miR-1182 expression was strikingly enhanced in Propofol-treated A2780 and SK-OV-3 cells, while miR-1182 expression was repressed in Propofol-treated cells transfected with anti-miR-1182 (Fig. 6C). In function, cell viability and colony formation were notably suppressed by Propofol, while further miR-1182 inhibition restored cell viability and colony formation ability (Fig. 6D and E). Besides, glucose consumption, lactate production and ATP level were strikingly inhibited by Propofol, while further miR-1182 deficiency restored glucose consumption, lactate production and ATP level in Propofol-treated A2780 and SK-OV-3 cells (Fig. 6F-H). In addition, cell migration and cell invasion were notably blocked in A2780 and SK-OV-3 cells treated with Propofol but partly recovered in Propofol-treated cells transfected with anti-miR-1182 (Fig. 6I-K). These data suggested that Propofol played functions by increasing the expression of miR-1182.

### MiR-1182 bound to S100B 3’UTR to inhibit S100B expression

**Fig. 7:**
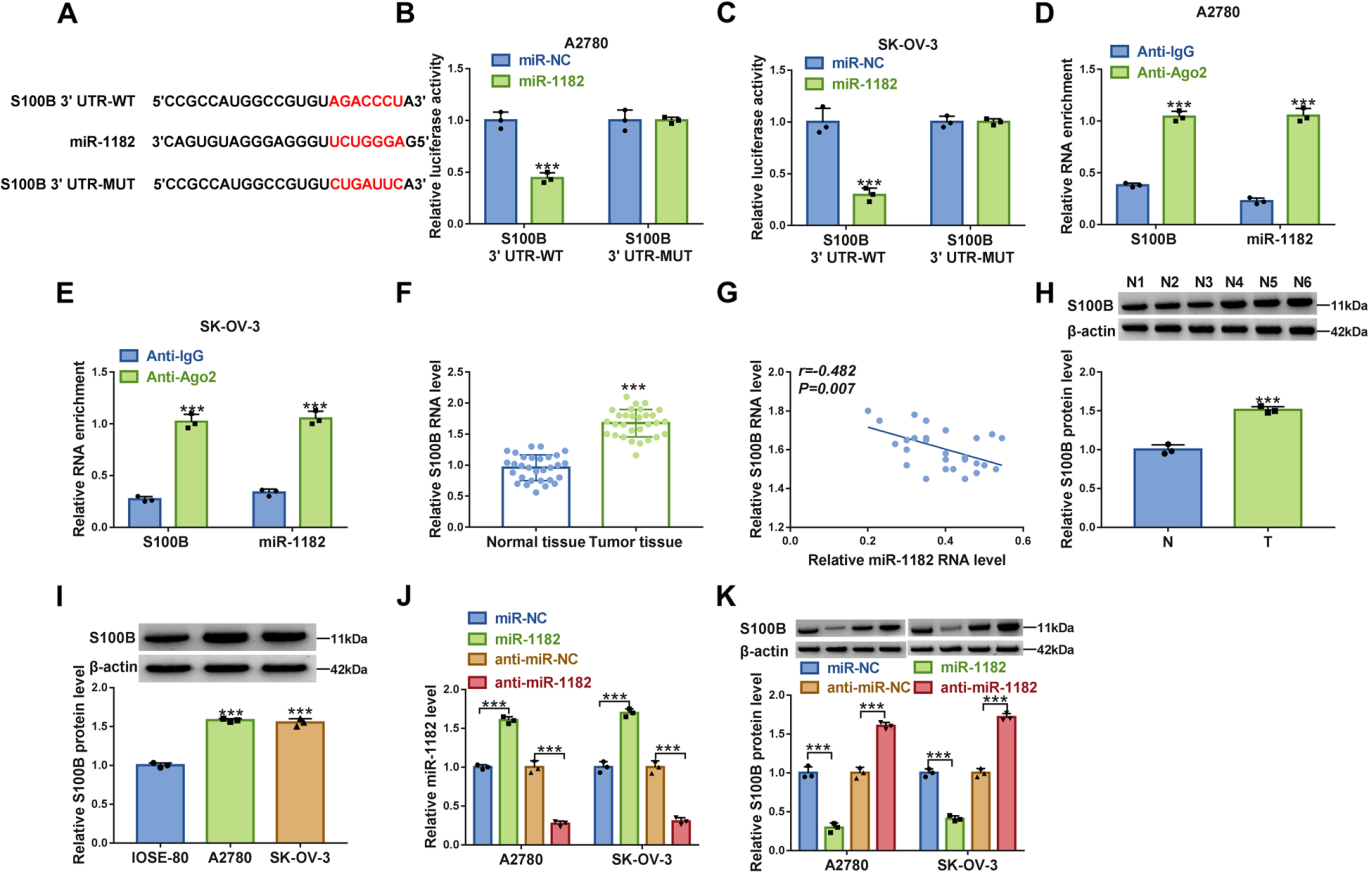
S100B was a target of miR-1182. **A** The relationship between miR-1182 and S100B was predicted by Targetscan. **B** and **C** The relationship between miR-1182 and S100B was validated by dual-luciferase reporter assay. **D** and **E** The relationship between miR-1182 and S100B was validated by RIP assay. **F** The expression of S100B in tumor tissues and normal tissues was checked by qPCR. **G** The correlation between S100B expression and miR-1182 expression in tumor tissues. **H** The expression of S100B in tumor tissues was detected by western blot. **I** The expression of S100B in IOSE-80, A2780 and SK-OV-3 cells was detected by western blot. **J** The efficiency of miR-1182 mimic and inhibitor was checked by qPCR. **K** The effects of miR-1182 enrichment or inhibition on S100B protein level were determined by western blot. ***P < 0.001

To explore the regulatory network of miR-1182, the downstream targets of miR-1182 were identified. As predicted by Targetscan, miR-1182 directly bound to S100B 3’UTR via a special binding site (Fig. 7A). Dual-luciferase reporter assay exhibited that luciferase activity was notably decreased in A2780 and SK-OV-3 cells transfected with miR-1182 and S100B 3’UTR-WT (Fig. 7B and C). RIP assay showed that both miR-1182 and S100B could interact with Ago2 protein but not IgG protein (Fig. 7D and E). The expression of S100B mRNA was strikingly increased in tumor tissues compared with that in normal tissues (Fig. 7F), and S100B mRNA expression was negatively correlated with miR-1182 expression in tumor tissues (Fig. 7G). The protein level of S100B was also higher in tumor tissues than that in normal tissues (Fig. 7H). The expression of S100B protein was also higher in A2780 and SK-OV-3 cells than that in IOSE-80 cells (Fig. 7I). The expression of miR-1182 was enhanced in A2780 and SK-OV-3 cells transfected with miR-1182 compared to miR-NC, while the expression of miR-1182 was impaired in cells transfected with anti-miR-1182 compared to anti-miR-NC (Fig. 7J). Besides, miR-1182 overexpression markedly inhibited S100B expression, and miR-1182 deficiency promoted S100B expression (Fig. 7K). These data suggested that miR-1182 bound to S100B and thus inhibited S100B expression.

### S100B overexpression partly abolished the effects of Propofol

**Fig. 8:**
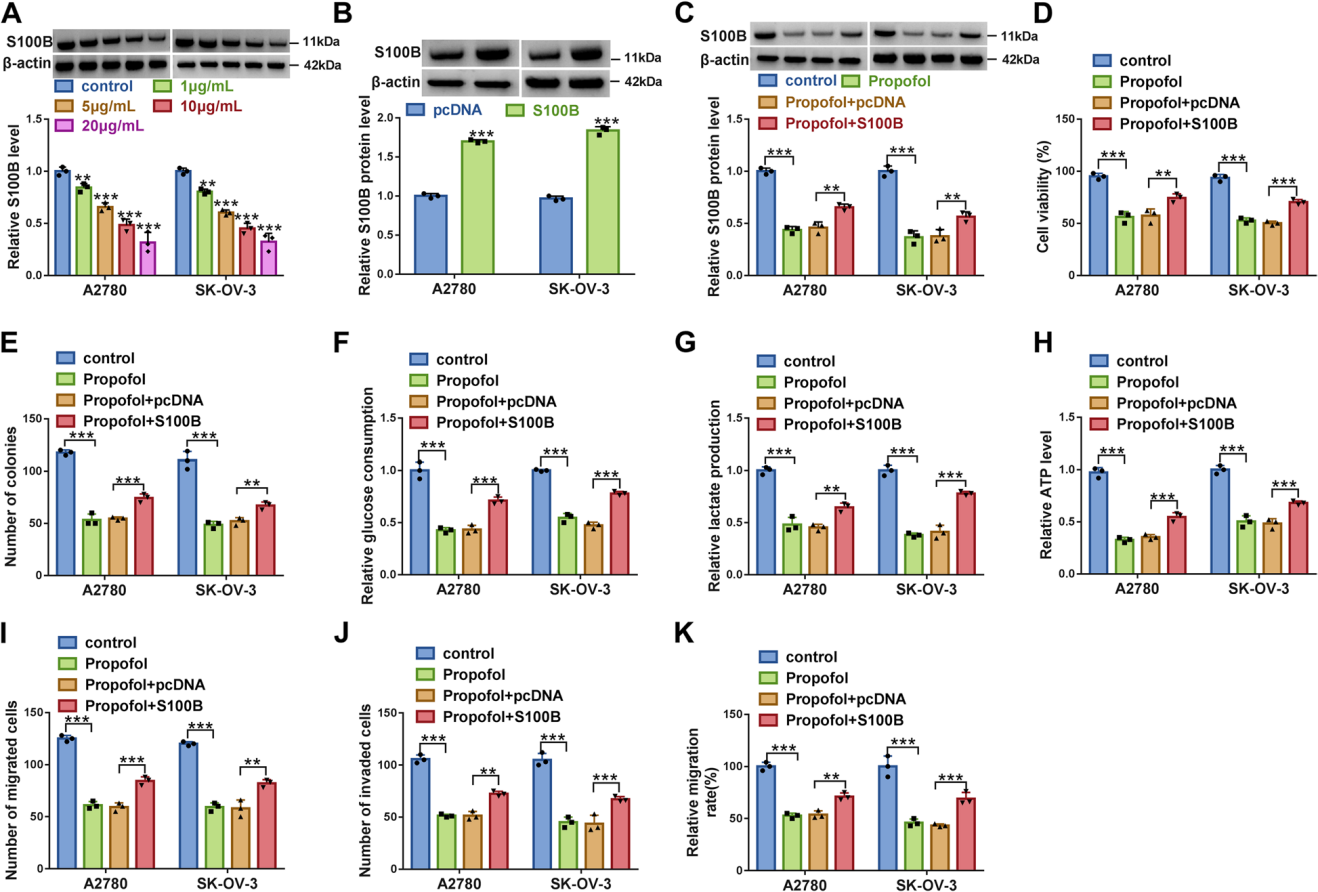
Propofol blocked A2780 and SK-OV-3 cell malignant behaviors by sequestering S100B. **A** The protein level of S100B in different doses of Propofol-treated A2780 and SK-OV-3 cells was checked by western blot. **B** The efficiency of S100B overexpression was checked by western blot. Then, in Propofol-treated A2780 and SK-OV-3 cells transfected with S100B or pcDNA, **C** the protein level of S100B was checked by western blot. **D** Cell viability was detected by MTT assay. **E** Colony formation ability was detected using colony formation assay. **F**-**H** Glucose consumption, lactate production and ATP level were investigated using matched kits. **I**-**K** Cell migration was assessed by transwell assay and wound healing assay, and cell invasion was assessed by transwell assay. **P < 0.01 and ***P < 0.001

The data from western blot manifested that the expression of S100B was strikingly decreased in Propofol-treated A2780 and SK-OV-3 cells in a dose-dependent manner (Fig. 8A). The efficiency of S100B overexpression was examined, and we found that the protein level of S100B was remarkably enhanced in A2780 and SK-OV-3 cells transfected with S100B compared to pcDNA (Fig. 8B). The expression of S100B was suppressed in Propofol-treated A2780 and SK-OV-3 cells, while S100B expression was recovered in Propofol-treated A2780 and SK-OV-3 cells transfected with S100B (Fig. 8C). In function, Propofol-suppressed cell viability and colony formation ability were largely recovered by further S100B reintroduction (Fig. 8D and E). Glucose consumption, lactate production and ATP level were depleted in Propofol-treated A2780 and SK-OV-3 cells but partly restored in Propofol-treated cells transfected with S100B (Fig. 8F-H). Moreover, Propofol-inhibited cell migration and cell invasion in A2780 and SK-OV-3 cells were partly promoted by S100B reintroduction (Fig. 8I-K). These data suggested that Propofol inhibited ovarian cancer cell malignant behaviors by depleting S100B expression.

### Circ_MUC16 inhibited miR-1182 expression to promote S100B level

**Fig. 9:**
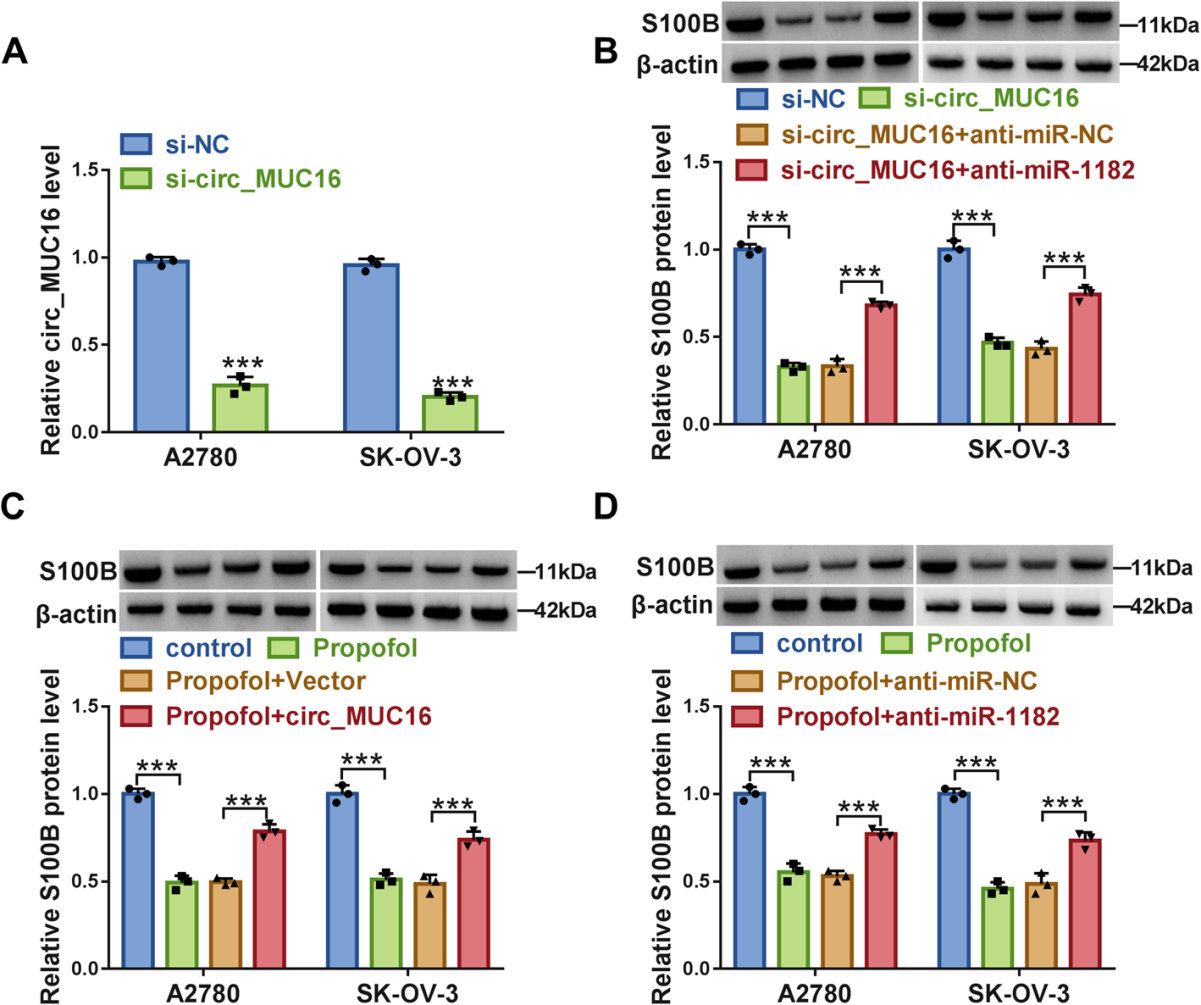
Circ_MUC16 acted as miR-1182 sponge to suppress miR-1182 expression and thus promote S100B level. **A** The efficiency of circ_MUC16 interference was checked by qPCR. **B** The protein level of S100B in A2780 and SK-OV-3 cells transfected with si-circ_MUC16, si-NC, si-circ_MUC16 + anti-miR-1182 or si-circ_MUC16 + anti-miR-NC were examined by western blot. **C** The protein levels of S100B in Propofol-treated A2780 and SK-OV-3 cells transfected with circ_MUC16 or Vector were checked by western blot. **D**) The protein levels of S100B in Propofol-treated A2780 and SK-OV-3 cells ransfected with anti-miR-1182 or anti-miR-NC were checked by western blot. ***P < 0.001

The transfection of si-circ_MUC16 remarkably weakened the expression of circ_MUC16 in A2780 and SK-OV-3 cells (Fig. 9A). Additionally, we discovered that the protein level of S100B was remarkably decreased in A2780 and SK-OV-3 cells transfected with si-circ_MUC16 alone, while the cotransfection of si-circ_MUC16 and anti-miR-1182 partly recovered the expression of S100B (Fig. 9B). Besides, in Propofol-treated A2780 and SK-OV-3 cells, the inhibitory expression of S100B protein was largely recovered by circ_MUC16 overexpression or miR-1182 inhibition (Fig. 9C and D). The data suggested that circ_MUC16 acted as the sponge of miR-1182 to inhibit miR-1182 expression and thus promote the expression of S100B.

## Discussion

Increasing studies have shown that Propofol inhibits proliferation, migration, and invasion of various tumor cells by regulating the expression of numerous signaling pathways and ncRNAs [19]. This is crucial for selecting suitable anesthetic drugs for different types of cancer to achieve the best outcomes for cancer patients. Our data manifested that circ_MUC16 expression was significantly elevated in ovarian cancer, and Propofol treatment notably depleted circ_MUC16 expression in ovarian cancer cells. Besides, Propofol-suppressed proliferation, migration and invasion in ovarian cancer cells were recovered by circ_MUC16 overexpression. Our study proposed that circ_MUC16 aggravated ovarian cancer development by reinforcing S100B expression via competitively binding to miR-1182. Our study, for the first time, defined the function of circ_MUC16 responding to Propofol in ovarian cancer, and forced expression of circ_MUC16 might attenuate the effects of Propofol in ovarian cancer.

Circ_MUC16 has been rarely investigated in human cancers, and its functions are largely unclear. Gan et al. showed that circ_MUC16 expression was significantly increased in epithelial ovarian cancer tissues by RNA sequencing [7]. Their functional assays revealed that circ_MUC16 promoted autophagy and thus enhanced invasion and metastasis of epithelial ovarian cancer [7]. Consistent with these data, our study showed that circ_MUC16 silencing suppressed proliferation, migration and invasion in ovarian cancer cells in vitro, and circ_MUC16 knockdown also attenuated tumor growth in vivo. These findings maintained the oncogenic role of circ_MUC16. Besides, we found that Propofol depleted the expression of circ_MUC16 and thus inhibited ovarian cancer malignant behaviors in vitro and in vivo, which might be a crucial way for Propofol to play its effects.

Numerous studies demonstrated that circRNA regulated multiple biological functions in human diseases even cancers by acting as miRNA sponges [25, 28, 7]. Our data presented that circ_MUC16 was mainly located in the cytoplasm of A2780 and SK-OV-3 cells. We speculated that circ_MUC16 might play a competing endogenous RNA (ceRNA) mechanism to compete with certain mRNAs for miRNAs. With the help of bioinformatics tools, several miRNAs targeted by circ_MUC16 were predicted. MiR-1182 was screened and validated by dual-luciferase reporter assay and RIP assay. Besides, a previous study held the view that miR-1182 was downregulated in ovarian cancer tissues and cells, and miR-1182 upregulation decelerated ovarian cancer cell proliferation, migration and invasion [9]. Consistently, miR-1182 was shown to block the development of gastric cancer and bladder cancer [23, 27]. In agreement with these data, our rescue experiments presented that miR-1182 deficiency partly abolished the effects of circ_MUC16 silencing and then recovered ovarian cancer cell proliferation, migration and invasion, while miR-1182 restoration inhibited these malignant phenotypes.

S100B is one of the members of the S100 protein family and is involved in the progression of a variety of cancers [3, 16]. Previous studies showed that the expression of S100B was strikingly enhanced in ovarian cancer tissues [20], and S100B overexpression promoted ovarian cancer stem cell chemoresistance and stemness [20, 21]. The cancer-promoting effects of S100B were also declared in glioma, colon cancer and others [8, 16]. Consistent with these views, our data showed that S100B overexpression restored proliferation, migration and invasion in ovarian cancer cells that were suppressed by miR-1182 restoration, suggesting that S100B promoted ovarian cancer malignant development.

Noticeably, results from the present study cannot be extrapolated to the clinics. The benefits of using Propofol for patients with ovarian cancer are unknown, and the current cell culture conditions do not mimic the clinical use of Propofol. Therefore, the clinical application of Propofol in the treatment of ovarian cancer needs further validation.

## Conclusion

In summary, circ_MUC16 attenuates the effects of Propofol to promote the development of ovarian cancer by mediating the miR-1182/S100B signaling pathway. Our study deepens the understanding of Propofol functional mechanism in ovarian cancer, and the data strongly support that Propofol plays effects by mediating the expression of circRNAs.

## Supplementary Information

**Additional file 1.**

## Data Availability

All data generated or analyzed during this study are included in this article.

## Abbreviations

ncRNAs: non-coding RNAs
miR: microRNA
circ_MUC16: Circular RNA mucin 16
S100B: S100 calcium-binding protein B
qPCR: Quantitative real-time polymerase chain reaction
MTT: 3-(4, 5-di methyl thiazol-2-yl)-2, 5-di phenyl tetrazolium bromide
DMEM: Dulbecco’s Modified Eagle Medium
RIP: RNA immunoprecipitation
RIPA: Radioimmunoprecipitation assay
BCA: Bicinchoninic Acid
SDS-PAGE: Sodium dodecyl sulfate-polyacrylamide gel electrophoresis
PVDF: Polyvinylidene fluoride
