# Supplementary figures and images for: Circ_MUC16 attenuates the effects of Propofol to promote the aggressive behaviors of ovarian cancer by mediating the miR-1182/S100B signaling pathway
Source: BMC Anesthesiol. 2021 Nov 27;21:297. doi: 10.1186/s12871-021-01517-0 (PMC8626908; doi:10.1186/s12871-021-01517-0)

FIG7H

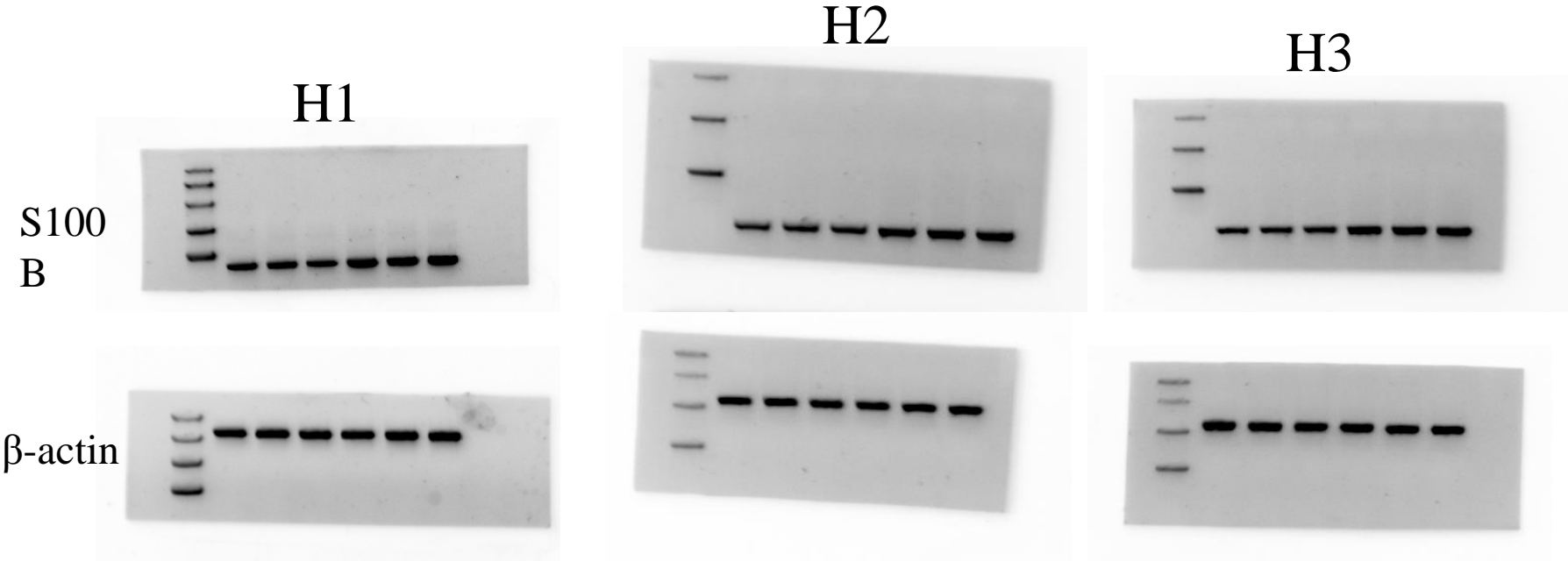

FIG7I

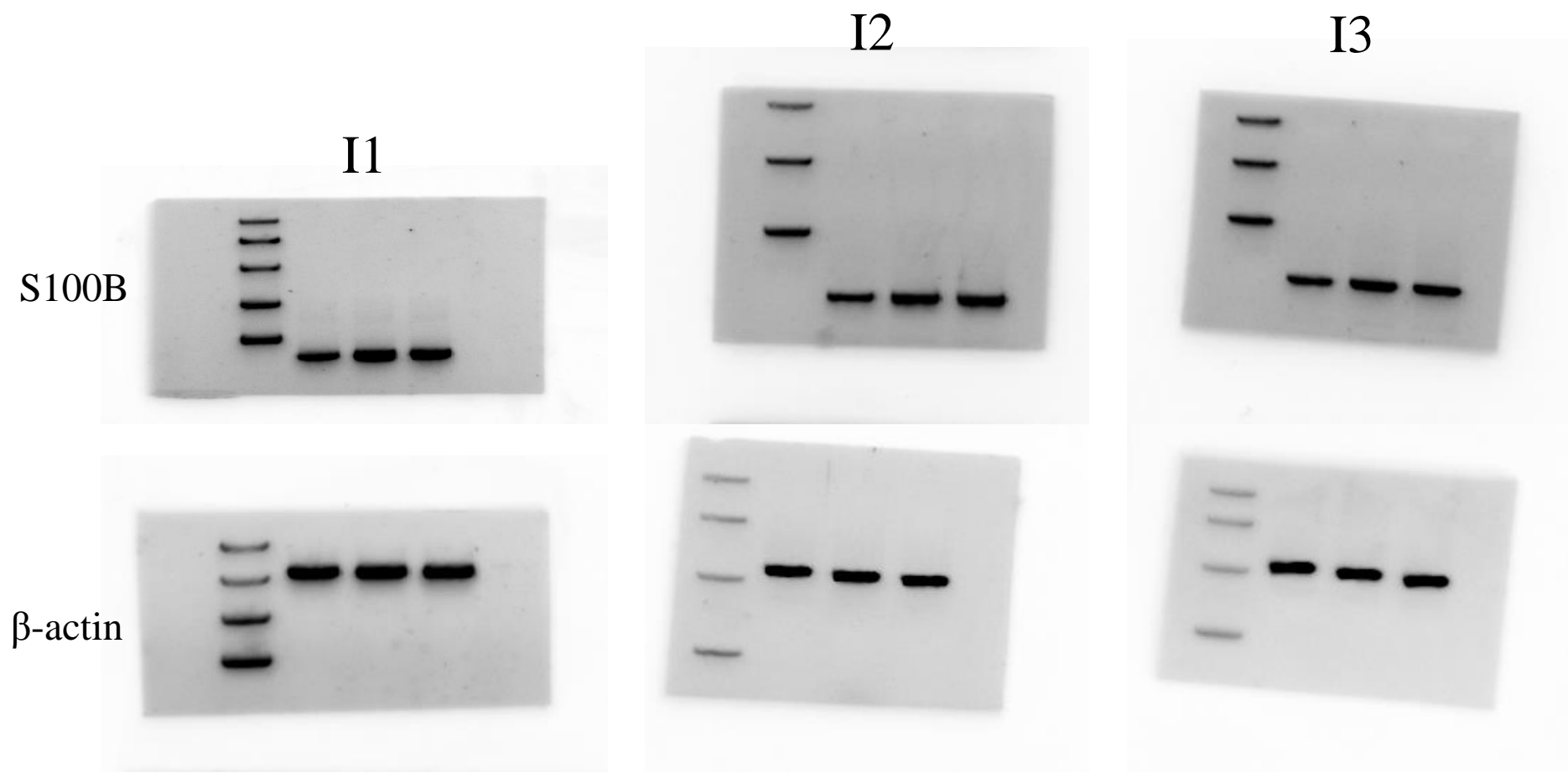

FIG7K

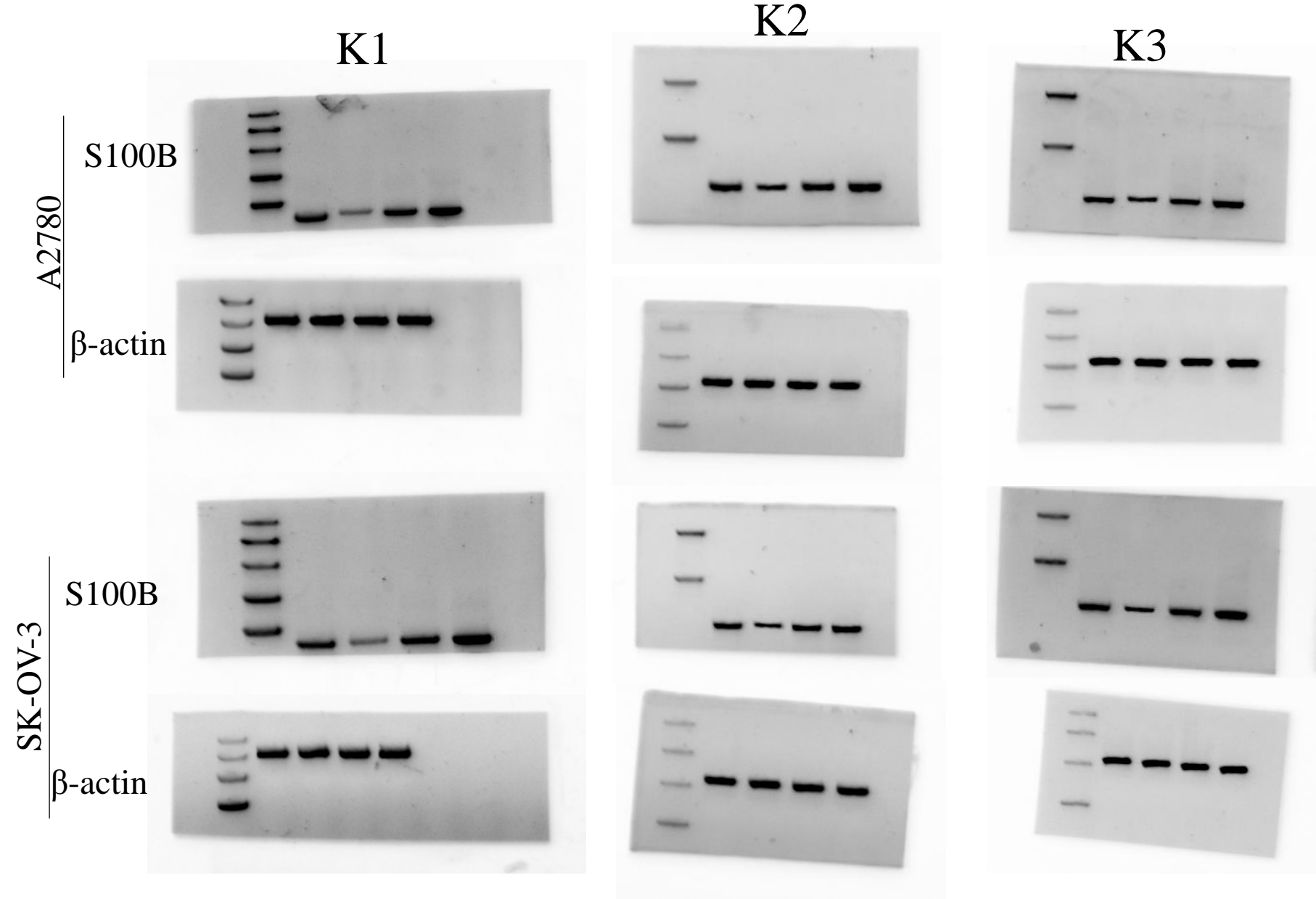

FIG8A

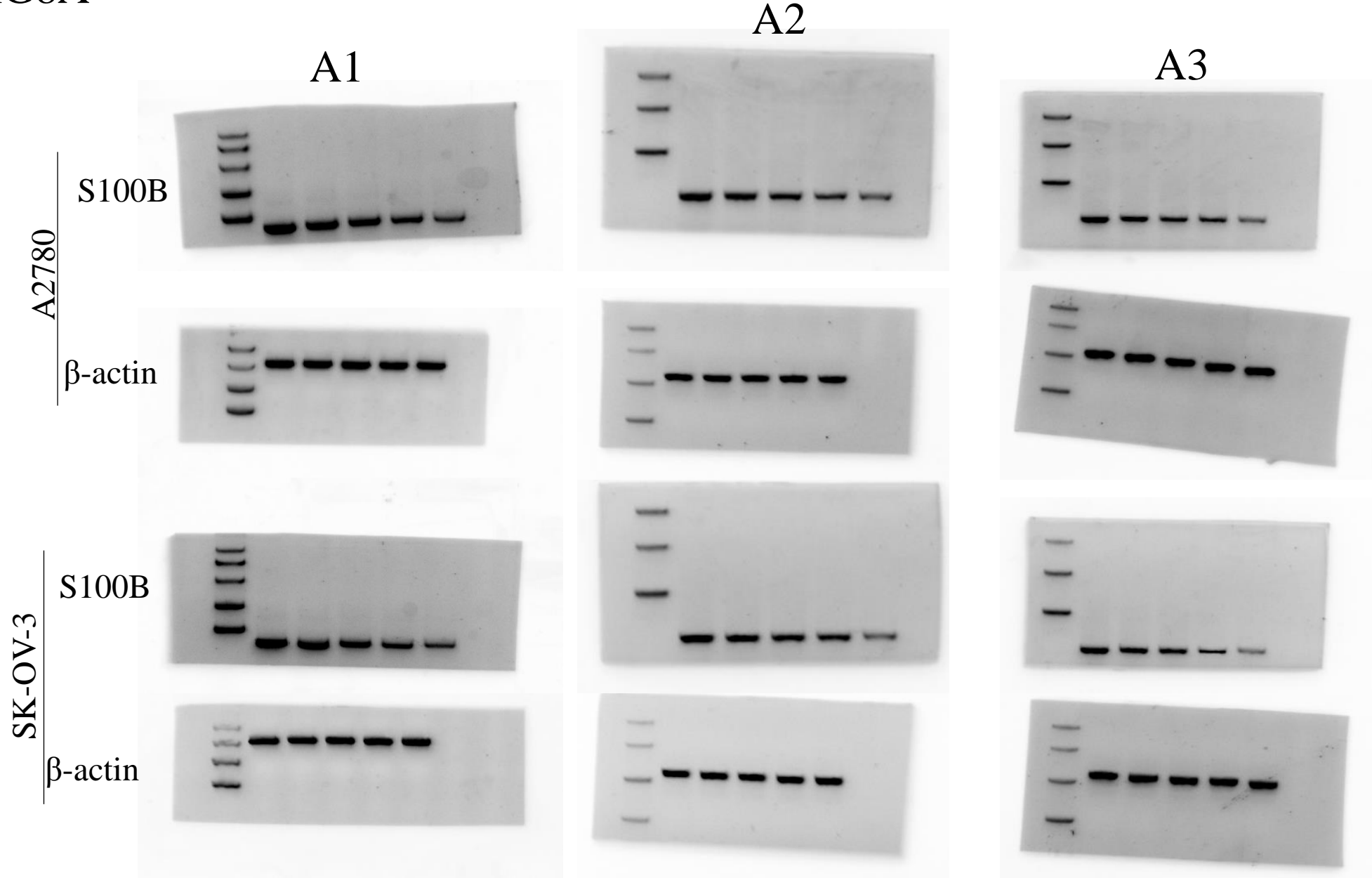

FIG8B

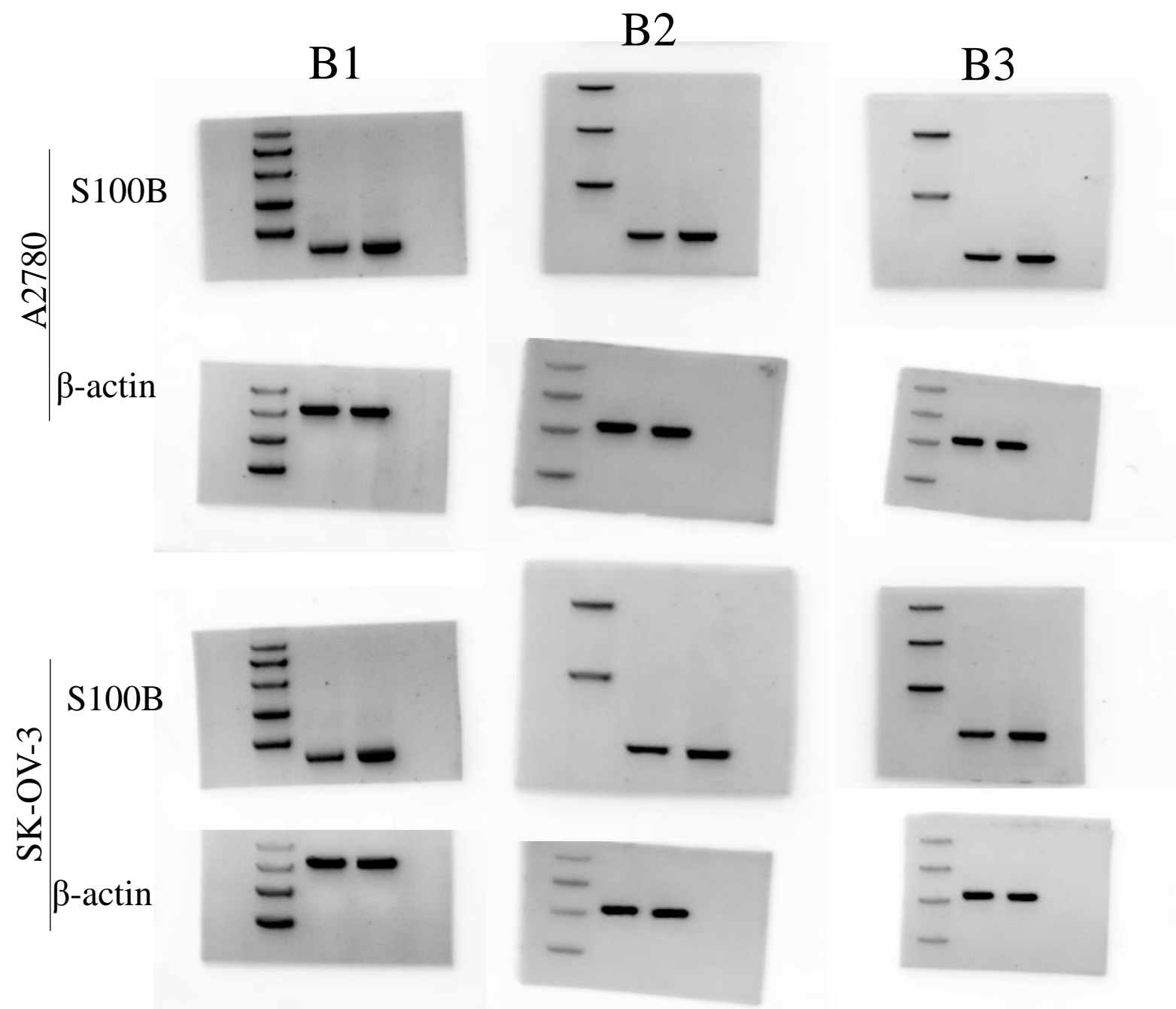

FIG8C

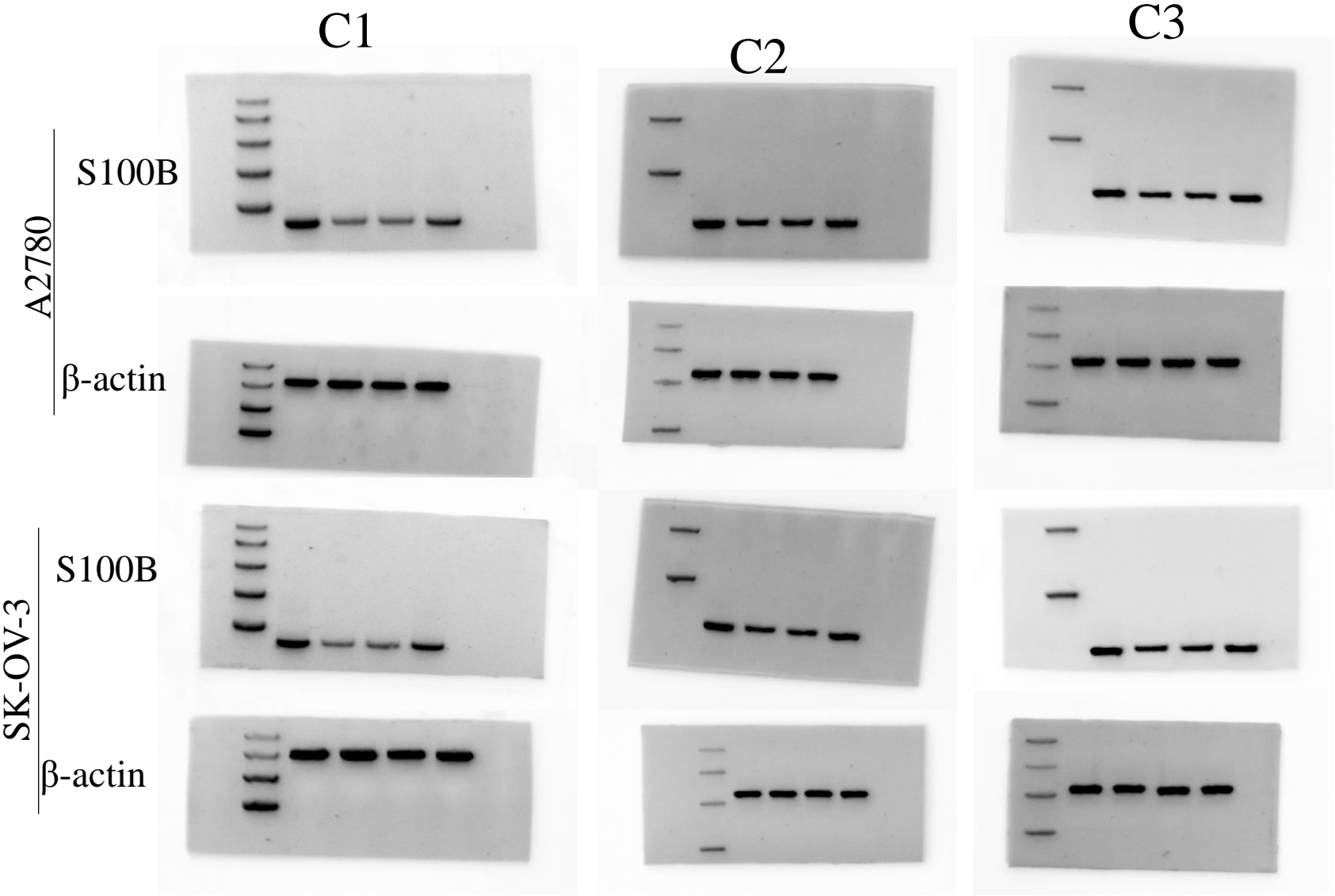

FIG9B

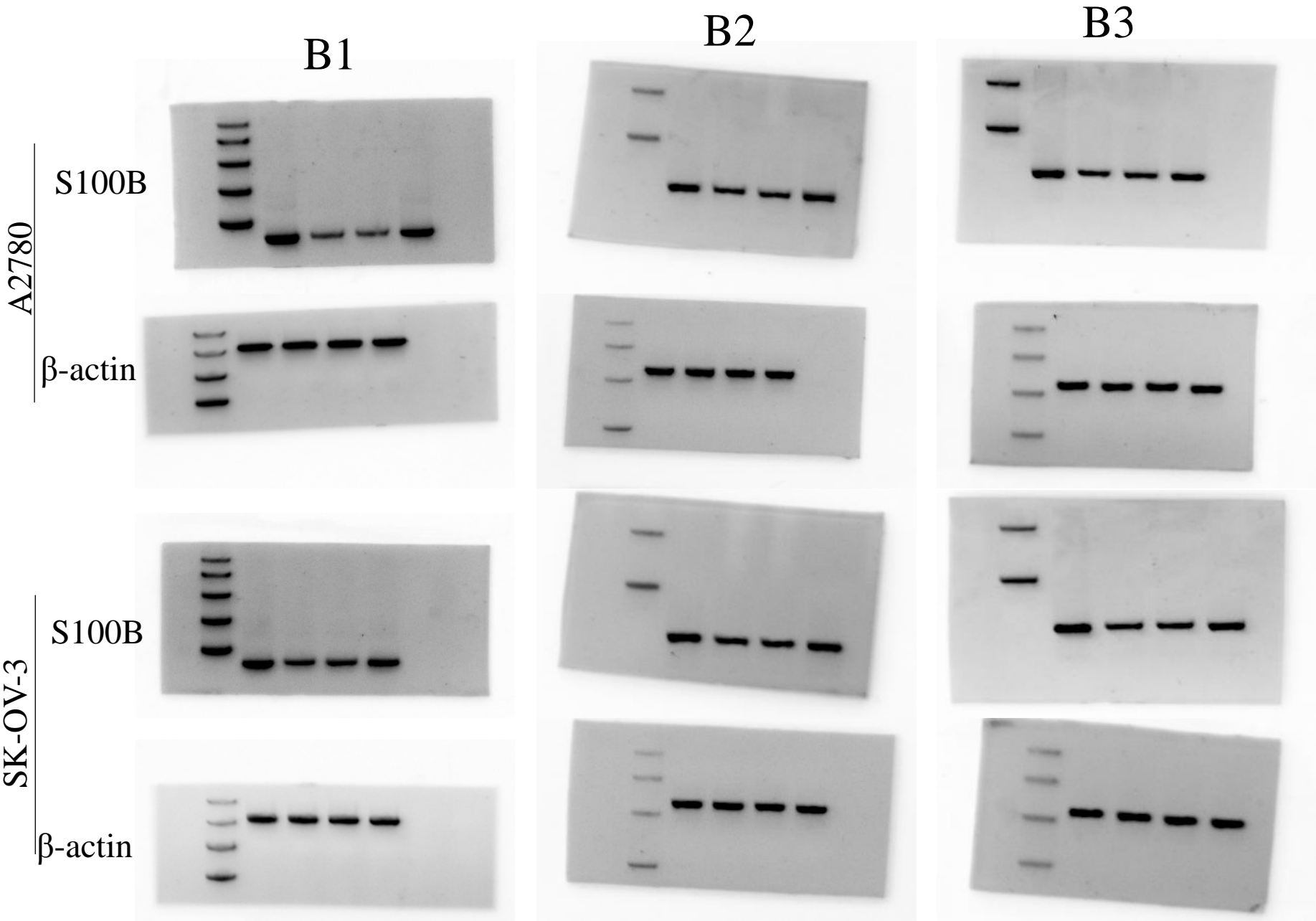

FIG9C

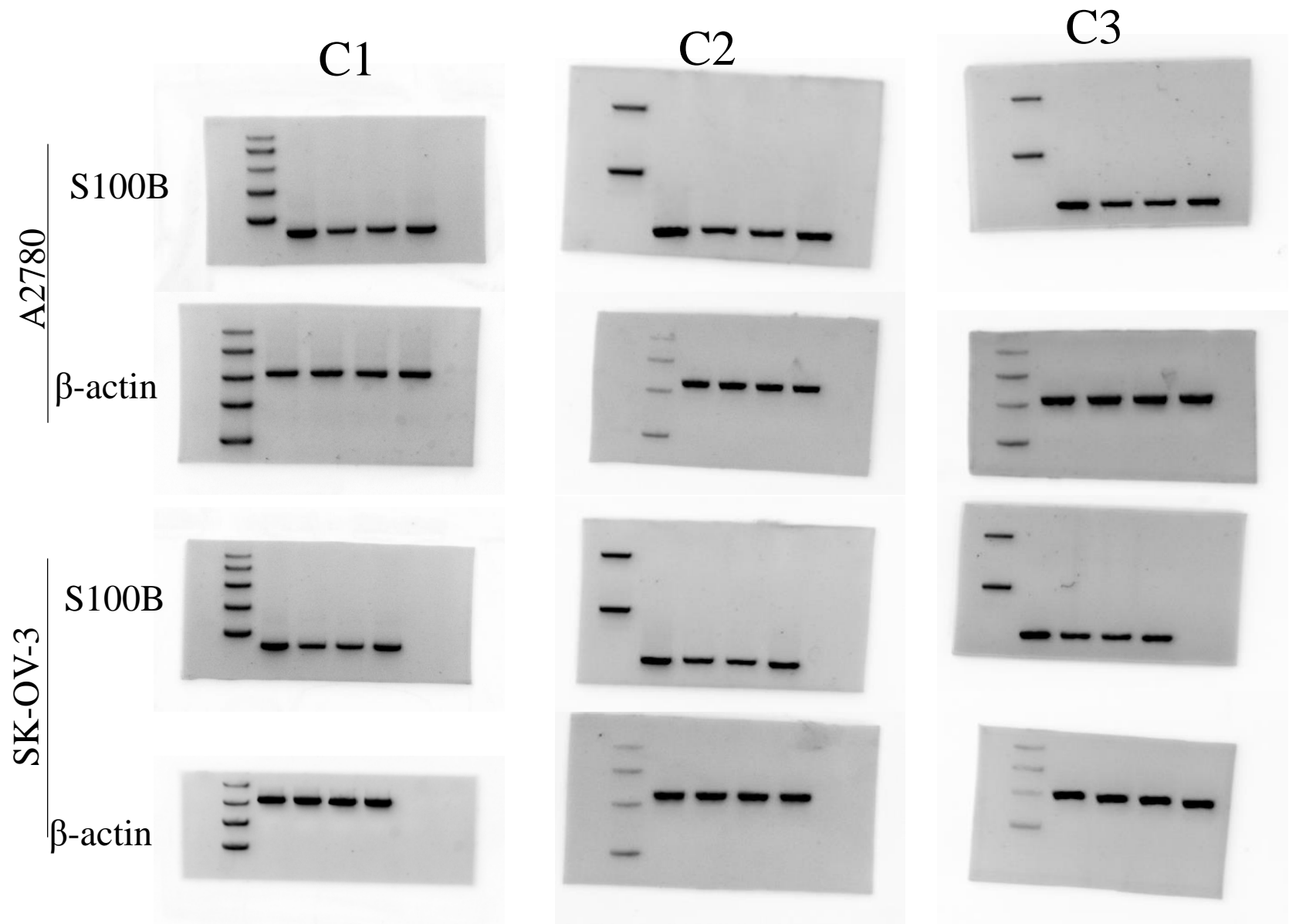

FIG9D

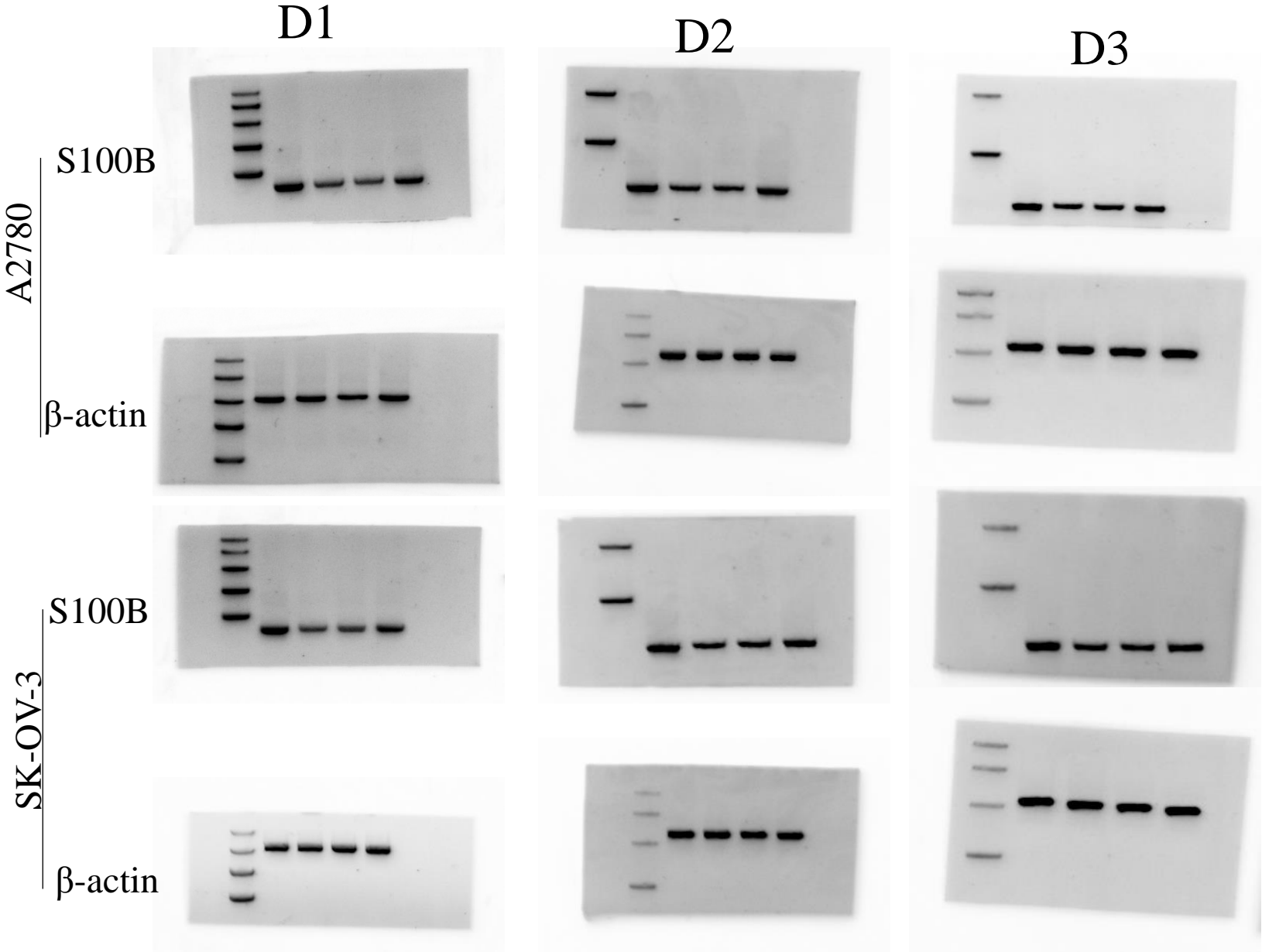

Supplement: Supplementary file 1 — Additional file 1. [file 12871_2021_1517_MOESM1_ESM.pdf]
